# IUC24421-78 Treatment timing and chronobiological aspects of ADT resistance in real-world mCRPC patients

**DOI:** 10.1093/oncolo/oyaf248.037

**Published:** 2025-08-29

**Authors:** I Bivolarski

**Affiliations:** Burgas, Bulgaria

## Abstract

**Background:**

Timing may represent an overlooked variable in the treatment of metastatic castration-resistant prostate cancer (mCRPC), where resistance to androgen receptor (AR)-targeted therapies eventually emerges in all patients. The clinical trajectory of mCRPC may be influenced by circadian factors known to affect endocrine sensitivity, DNA repair pathways, and drug metabolism. Preclinical evidence suggests that AR signaling itself may be modulated by circadian regulators, raising the possibility that treatment timing could impact therapeutic outcomes. To examine the relationship between the timing of ADT, AR-targeted agents, or docetaxel administration and time to progression or overall survival in a real-world cohort of patients with mCRPC.

**Methods:**

We retrospectively analyzed data from 25 patients with mCRPC treated at a single oncology center between 2024 and 2025. Data were extracted from electronic medical records and verified by physician review. The timing of drug administration (morning vs. afternoon) was determined based on infusion schedules recorded in patient charts. Variables included type of therapy (ADT, AR-targeted agents, or docetaxel), time to progression (TTP), and survival status. Patients were grouped based on consistent morning (before 12:00) or afternoon (after 12:00) dosing.

**Results:**

Among 16 patients who received therapy consistently in the morning, a greater proportion demonstrated longer survival compared to 11 patients treated in the afternoon. Median TTP was 11.2 months in the morning group versus 6.3 months in the afternoon group. The trend was observed across both hormonal and chemotherapeutic agents when analyzed descriptively. No formal statistical testing was performed due to the limited cohort size.

**Conclusions:**

Patients receiving morning administration of ADT, AR-targeted agents, or docetaxel demonstrated a numerically longer time to progression and greater survival compared to those treated in the afternoon. Although preliminary and observational, these findings raise the possibility that treatment timing may influence outcomes in mCRPC. Further investigation in larger, time-stratified cohorts is warranted.

